# Epithelial-Mesenchymal Transition and Metastasis under the Control of Transforming Growth Factor β

**DOI:** 10.3390/ijms19113672

**Published:** 2018-11-20

**Authors:** Yutaro Tsubakihara, Aristidis Moustakas

**Affiliations:** 1 Department of Medical Biochemistry and Microbiology, Science for Life Laboratory, Uppsala University, Box 582, SE-751 23 Uppsala, Sweden; yutaro.tsubakihara@imbim.uu.se; 2 Ludwig Institute for Cancer Research, Biomedical Center, Uppsala University, Box 595, SE-751 24 Uppsala, Sweden

**Keywords:** epithelial-mesenchymal transition, micro-RNA, non-coding RNA, signal transduction, transcription factor, transforming growth factor β, tumor invasiveness

## Abstract

Metastasis of tumor cells from primary sites of malignancy to neighboring stromal tissue or distant localities entails in several instances, but not in every case, the epithelial-mesenchymal transition (EMT). EMT weakens the strong adhesion forces between differentiated epithelial cells so that carcinoma cells can achieve solitary or collective motility, which makes the EMT an intuitive mechanism for the initiation of tumor metastasis. EMT initiates after primary oncogenic events lead to secondary secretion of cytokines. The interaction between tumor-secreted cytokines and oncogenic stimuli facilitates EMT progression. A classic case of this mechanism is the cooperation between oncogenic Ras and the transforming growth factor β (TGFβ). The power of TGFβ to mediate EMT during metastasis depends on versatile signaling crosstalk and on the regulation of successive waves of expression of many other cytokines and the progressive remodeling of the extracellular matrix that facilitates motility through basement membranes. Since metastasis involves many organs in the body, whereas EMT affects carcinoma cell differentiation locally, it has frequently been debated whether EMT truly contributes to metastasis. Despite controversies, studies of circulating tumor cells, studies of acquired chemoresistance by metastatic cells, and several (but not all) metastatic animal models, support a link between EMT and metastasis, with TGFβ, often being a common denominator in this link. This article aims at discussing mechanistic cases where TGFβ signaling and EMT facilitate tumor cell dissemination.

## 1. Introduction

**Figure 1:**
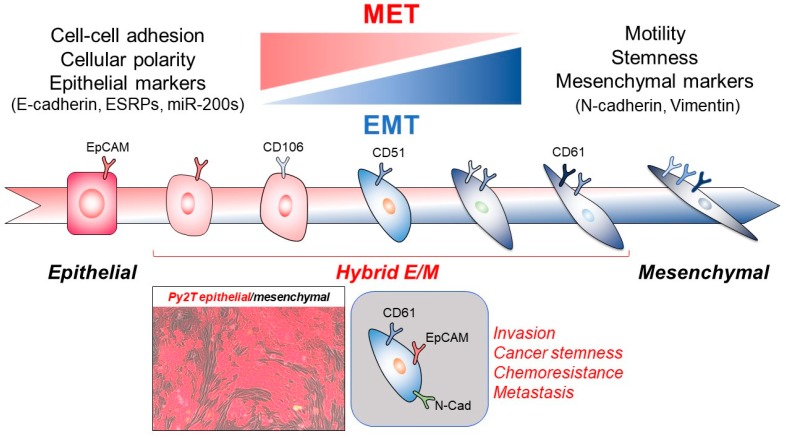
The trajectory of differentiation change is pictorially graphed with epithelial cells on the left, hybrid E/M cells in the middle, and mesenchymal cells on the right hand of the arrow. The EMT and MET are also depicted as gradients of molecular and phenotypic change at the top of the figure and inside the main trajectory. Specific molecular and cellular attributes of epithelial and mesenchymal cells are listed on top of the relevant cell types. Important cell surface antigens are also drawn on the plasma membrane of each cell in a different color, in order to mark the molecular progression from an epithelial to a mesenchymal phenotype and the intermediate stages. At the bottom, the photomicrograph shows a mixed population of epithelial (red) and mesenchymal (dark grey) Py2T mouse breast cancer cells that have undergone EMT followed by MET. An additional cell model depicts a possible hybrid E/M cell that expresses EpCAM, CD61, and N-cadherin (N-Cad) as revealed in certain studies of circulating tumor cells. The features of EMT that are relevant to cancer are listed on the right.

Epithelial cells differentiate from stem cells in various tissues and retain the ability to change their differentiation into different lineages, including a transition towards the mesenchymal lineage, best known as the epithelial-mesenchymal transition (EMT; Figure 1) [1,2]. An important aspect in the understanding of EMT has been the transitory nature of this process, which has also been termed plasticity, and naturally implies the ability of mesenchymal cells to undergo an inverse process, the mesenchymal-epithelial transition (MET; Figure 1) [1,2,3]. In fact, several studies on EMT have proposed the term metastable EMT or hybrid epithelial-mesenchymal intermediate phenotype in order to emphasize the generation of intermediate semi-differentiated forms of cells that can be generated either by epithelial or mesenchymal differentiated cells of origin (Figure 1) [4,5,6,7]. The architectural and cell biological similarity between epithelial and endothelial cells of the blood or lymphatic vessels also explains why endothelial cells can undergo endothelial-mesenchymal transition (EndMT) [8,9], however, the contribution of mesenchymal cells towards the generation of endothelial cells is a process with few clearly reported cases stemming from organ development, such as the heart and mesenchymal tumors, i.e., sarcomas [10,11]. These latter aspects will not be discussed further.

Under most EMT processes in cancer, epithelial cells generate newly synthesized extracellular matrix (ECM) glycoproteins and corresponding cell surface receptors that communicate with the new ECM, generating a new cellular microenvironment that enforces further changes in cell differentiation [1]. Concomitantly, changes in cell-cell adhesion complexes (Figure 1), rendering them looser and more flexible, permit the mobility of single or cohorts of a finite number of tumor cells away from the differentiated epithelium [1]. Changes in cytoskeletal components (microfilaments, intermediate filaments, or even microtubules) drive the disassembly-reassembly of cell-cell contacts and also regulate cell motility (Figure 1), the cell cycle, gene expression, and new exocytosis, contributing to ECM neo-synthesis [1]. Under the MET, similar but not exactly the same reverse phenomena take place, but in general, re-building of epithelial cell-cell contacts (e.g., adherens and tight junctions) and supporting acto-myosin reassembly is a hallmark response of the MET (Figure 1) [5,6].

These changes in cellular architecture and function are usually initiated in response to many growth factors and cytokines. These, sometimes, are induced after the DNA damage response or after the sustained activity of dominant oncogenes that operate in tumor cells. The transforming growth factor β (TGFβ), receptor tyrosine kinase (RTK), the Wnt, Notch, hedgehog, hippo, the cytokine, and nuclear receptor pathways have all been implicated in the onset of EMT [12,13]. Members of the same signaling pathways can also initiate the METs during cancer progression [5,6]. A common feature of the EMT in a large variety of carcinomas is that many of the above signaling pathways crosstalk and share inter-connected regulatory components that play critical roles in the establishment of the EMT or MET. For example, TGFβ induces the expression of many other growth factors and cytokines, which further propagate their signaling activities, while also cooperating with the initial stimulus of TGFβ, generating positive feed-forward loops necessary for sustained signaling that supports the long process of EMT [3,14].

Growth factor and cytokine signaling pathways naturally function by regulating gene expression or by modulating enzymatic activities, processes of degradation or stability and processes of multi-molecular assembly in the cytoplasm, plasma membrane or specific organelles. Among the large number of molecular mechanisms, transcription factors (TF) that promote the EMT (EMT-TFs) have been extensively studied and contributed greatly to the understanding of this process [1]. EMT-TF activity is also coupled to extensive chromatin modification changes, often referred to as epigenetic remodeling, thus contributing to gene regulation, whereas specific splicing factors can also mediate the phenotypic changes outlined above that occur during EMT or MET [3,6]. The families of EMT-TFs include the proteins known as Snail1, Snail2/Slug, zinc finger E-box binding homeobox 1 and 2 (ZEB1 and ZEB2), and members of the Twist family [1,6]. These transcription factors have distinct and characteristic DNA-binding or transactivation domains, e.g., zinc-fingers for Snail1, Snail2, both zinc finger and homeodomain for ZEB1/2 and a basic helix loop helix for Twist1-3, acting as DNA-binding domains, yet they seem to regulate a similar set of genes and clearly contribute to the same overall phenotypic process, the EMT.

## 2. Regulation of EMT by TGF-β

**Figure 2:**
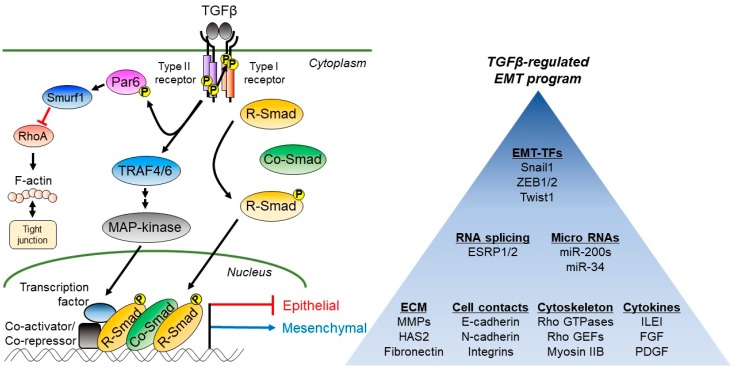
Basic TGFβ signaling diagram along with a program of TGFβ-regulated genes that contribute to the EMT. Left side, the extracellular dimeric TGFβ ligand is shown to bind to its plasma membrane receptors, the type II and type I receptors (each drawn as a dimer), causing trans-phosphorylation (circled P) of the type I receptor by the type II receptor. The type II receptor phosphorylates the polarity protein Par6, which recruits the ubiquitin ligase Smurf1 and regulates RhoA-dependent actin assembly and tight junction disassembly. The type I receptor also recruits the ubiquitin ligases TRAF4 and TRAF6, which activate the MAP-kinase pathway by ubiquitination, leading to the transcription factor phosphorylation. The type I receptor kinase also phosphorylates R-Smads, which form complexes with the Co-Smad, Smad4. In the nucleus, Smad complexes and cooperating transcription factors bound to various genes, along with co-repressors (co-activators) either repress the expression of epithelial genes or induce the expression of mesenchymal genes. Right side, a summary of the TGFβ-regulated EMT program divided into seven subprograms, each enlisting only a small representative example of genes that are involved in the EMT and cell motility. Signaling flow is indicated by black arrows (positive flow) and red T-bars (negative regulation); on the gene transcriptional start site, a red T-bar indicates negative regulation and a blue arrow indicates positive regulation of transcription.

Among the plethora of signaling pathways that induce the EMT in cancer, TGFβ has been shown to play prominent roles. The term TGFβ encompasses a large family of secreted polypeptides with diverse developmental and pathophysiological roles [15]; here we mainly refer to the three TGFβ isoforms, TGFβ1, TGFβ2, and TGFβ3. However, it is worth noting that other family members such as activins, nodal, or bone morphogenetic proteins (BMPs) can regulate either EMT or MET in the context of cancer. We will unfortunately not expand on these alternative growth factors and their mechanisms of action. The TGFβ family members are dimeric and disulfide-linked extracellular proteins deposited in the ECM of many tissues, including calcified bone, or in the secretory granules of various cells, including platelets, as latent, inactive proteins [16]. Mature TGFβ ligands are generated when various cell types get activated in the tissue microenvironment, and upon secretion of specific proteases and subsequent expression of integrin receptors, they enforce the chemical cleavage and conformational distortion of the latent ligands [17]. The active dimeric TGFβ can associate with transmembrane receptors known as type II and type I TGFβ receptors that act as protein kinases and phosphorylate cytoplasmic proteins on serine, threonine and less frequently on tyrosine (Figure 2) [13]. TGFβ ligand, the dimeric type II receptor together with the dimeric type I receptor form hetero-hexameric complexes [13]. The ligand-receptor signals via assembly of adaptor and regulatory proteins on the receptors (e.g., the recruitment and activation of tumor necrosis factor α receptor-associated factor (TRAF) ubiquitin ligases); or via the direct phosphorylation of Smad proteins catalyzed by the type I receptor or via the direct phosphorylation of polarity regulators on the tight junctions of epithelial cells, such as partitioning-defective 6 (Par6), catalyzed by the type II receptor (Figure 2) [15]. Receptor-activated (R-) Smads, Smad2, and Smad3 oligomerize with Smad4 and bind to DNA motifs or to many transcription factors, inducing or repressing the expression of genes, which regulate among other cellular processes, the EMT (Figure 2) [14]. The TRAF and other adaptors lead to either the activation of mitogen-activated protein (MAP) kinases or translocation of the cytoplasmic portion of the type I receptor after proteolytic cleavage to the nucleus, eliciting, like the Smad complexes, gene responses that facilitate the EMT [18,19]. This central TGFβ signaling pathway can be negatively regulated by several mechanisms. The inhibitory (I-) Smads, Smad6, and Smad7, whose expression can be induced by TGFβ, prominently act and mediate ubiquitination and lysosomal degradation of the type I receptor or inactivate R-Smad/Smad4 complexes in the nucleus [15,16]. The I-Smads mediate recruitment of TRAF ubiquitin ligases to the type I receptor and induce MAP-kinase signaling prior to receptor degradation [18,20]. In this manner, TGFβ signaling acts on many cell types and, in the context of cancer, it can promote EMT and tumor metastasis [21].

The above brief summary emphasizes the general and uniform principles of EMT and their regulation by TGFβ signaling that apply to all carcinomas studied. In the following sections, specific examples of regulatory mechanisms and individual molecules will be discussed. Readers are advised to consider carefully each example as a unique case that applies to a specific tumor type (always a carcinoma). The combination of in vitro cell-based or in vivo mouse-based models does not necessarily imply that these mechanisms, or the function of a specific molecule, are universally applicable to all cases of observed EMT. The reason of the context-dependent mechanisms in the most part remains unclear and, as a result, we will not attempt to classify the mechanisms according to a hypothetical scheme, proposing that a given mechanism is general or applicable only to some specific experimental models studied. Furthermore, the relevance of in vivo or in vitro EMT-based studies to human cancer pathology requires further detailed analysis.

### 2.1. Regulation of ECM Gene Expression by TGFβ

The ECM changes characteristically during cancer progression. A stiffer ECM, enriched in collagen-based cables that surround the tumor cells and infiltrate the tumor microenvironment, cause a special histology of advanced carcinomas, known as desmoplasia (tissue with excessive, fibrotic extracellular matrix, appearing as connective tissue). TGFβ secreted by tumor cells, associated fibroblasts (cancer-associated fibroblast, CAFs), or immune cells, is capable of inducing new ECM synthesis, ECM remodeling based on the secretion of metalloproteases and phenotypic changes that promote cell invasion [22]. TGFβ, in a time- and dose-dependent manner can induce the expression and stability of several ECM components. Various ECM genes are regulated by the TGFβ pathway via Smad and MAP-kinase signaling (Figure 2). For example, TGFβ, via Smad, Jun N-terminal kinase (JNK), and p38 MAP-kinase can regulate the expression of MMP2, whereas, via TGFβ-activated kinase 1 (TAK1) signaling, the latter activating nuclear factor κB (NF-κB) induces MMP9 expression [23,24,25]. Based on such signaling mechanisms, the new ECM is composed of TGFβ-induced fibronectin (FN1), a rather universal response of tissues to TGFβ signaling, of new collagen isoforms, new matrix metalloproteases (MMPs, MMP2, and MMP9 being most representative), new integrin β receptor subunits, and increased levels of plasminogen activator inhibitor 1 (PAI-1), among other matrix constituents [23,26]. The coordinate regulation of MMP and integrin receptor expression is known to contribute to cell migration and the remodeling of the ECM that facilitates epithelial cell invasiveness through the basement membrane.

EMT promotes invasive cell behavior by generating invadopodia on the membrane of the transitioning mesenchymal cells, a process induced by platelet-derived growth factor (PDGF) signaling; Twist1 activation induces PDGF receptor α and mediates PDGF signaling and tumor cell invasiveness [27]. Proteome-wide studies have established a widespread impact of TGFβ signaling on ECM and cell surface receptor N-linked glycosylation [28]. The specific roles of protein-based glycosylation on mechanisms of EMT remain to be elucidated. Yet, one case feeds back to TGFβ signaling. This is based on the glycosylating enzymes fucosyl-transferase 3 and 6, which fucosylate the type I receptor of TGFβ, a modification shown to be required for the EMT response of colorectal cancer cells [29].

The newly synthesized ECM proteins provide cues for cell migration (e.g., fibronectin) but also have signaling capacity. ECM proteins stimulate integrin receptors on the membrane of cancer cells, promoting EMT during breast cancer cell invasion, interestingly, by controlling the release of the active TGFβ ligand [30]. In prostate cancer cells, ECM-deposited latent TGFβ can be activated by membrane type I-matrix metalloprotease (MT1-MMP/MMP14), which then induces the secretion of Wnt5a; the sequential action of TGFβ and Wnt5a sustain EMT and invasiveness of the tumor cells [31]. Not only glycoproteins, but also glycosaminoglycans, such as hyaluronan, can be induced by TGFβ signaling (Figure 2). Hyaluronan is thought to act as a co-factor in the ECM that facilitates the access of TGFβ to its receptor complex, thus activating the signaling and expression of Snail1 or Twist1, which promote EMT in cancer cells [32]. In this study, high hyaluronan level in the tumor microenvironment ECM was achieved by the transgenic expression of HAS2, the hyaluronan synthase 2 [32]. It is formally possible that HAS2 acts in a different manner as a signaling protein and its positive actions on EMT may not strictly require extracellular hyaluronan production. This hypothesis is supported by studies of EMT in breast epithelial cells, where TGFβ induces *HAS2* mRNA expression [33]. Extracellular hyaluronan degradation by hyaluronidase or the antibody-mediated block of the major hyaluronan receptor, CD44, failed to inhibit the HAS2-mediated EMT responses [33]. The interplay between ECM molecules and TGFβ is also confirmed by studies of the impact of ECM stiffness on TGFβ-induced EMT; the EMT required a stiff ECM, whereas a soft ECM led to epithelial cell death instead of the pro-survival signals that maintain the EMT [34]. This observation appears reasonable since TGFβ-activated Smad complexes interact with the transcriptional mediators Yes-associated protein (YAP)/transcriptional coactivator with a PDZ-binding domain (TAZ) (YAP/TAZ) of the Hippo pathway that responds to ECM stiffness, possibly via collagen-dependent plasma membrane receptors, thereby providing another crosstalk mechanism between TGFβ and another developmental pathway during the process of EMT [35].

### 2.2. Regulation of Cell Contacts by TGFβ Signaling

Loss of adherens junctions is a hallmark of EMT, and TGFβ can induce E-cadherin loss by transcriptional repression (that requires long-term sustained signaling) of the *E-cadherin* (*CDH1*) gene and lysosomal degradation of cell surface E-cadherin, a process contributing to breast cancer metastasis [36]. During EMT, the loss of E-cadherin is often accompanied by gain of N-cadherin (CDH2) expression, which provides new, more flexible cell-cell contacts that are compatible with cancer cell migration (Figure 2) [37]. Additional cadherins undergo expression changes during EMT, as for example is the TGFβ-induced expression of cadherin-11 (osteoblastic cadherin) in lung adenocarcinoma cells [38]. Another mesenchymal cell cadherin induced by TGFβ during the EMT is cadherin-6 (K-cadherin), which facilitates the invasiveness of thyroid cancer cells [39].

The EMT requires the disassembly of tight junctions, a process usually directly linked to the regulation of polarity complex components. Polarity proteins bind to the cytoplasmic domains of tight junction proteins and assist junctional assembly [40]. By inducing Snail1, TGFβ achieves downregulation of the polarity regulator Crumbs3 (CRB3), which initiates loss of tight junctions during EMT [41]. Via transcriptional induction of the staphylococcal nuclease and tudor domain containing 1 (SND1) co-activator, expression of the ubiquitin ligase Smad ubiquitylation regulatory factor 1 (Smurf1) is enhanced, causing the degradation of the RhoA small GTPase during EMT [42]. RhoA activity controls actin polymerization near the tight junctions and promotes tight junction assembly; RhoA degradation inversely promotes tight junction disassembly. This transcriptional mechanism is accompanied by a more direct signaling mechanism whereby the TGFβ type II receptor phosphorylates the polarity adaptor protein Par6, required for the binding of Smurf1 to Par6, which then ubiquitinates and degrades RhoA (Figure 2) [40]. The transcriptional and protein degradation-based mechanisms are further complemented with mechanisms that control mRNA stability and translation. The micro-RNA (*miR*)*-155* inhibits *RhoA* mRNA translation [43]. In a parallel manner, the partner of Par6 in the polarity complex, Par3, is translationally repressed by the *miR-491-5p*, whose expression is induced by TGFβ [44]. It is worth noting here that the regulation of Par6/RhoA by the TGFβ type II receptor and Smurf1 is coupled to the ubiquitination and lysosomal degradation of the TGFβ receptor complex by coordinate actions of Smurf1 and Smurf2 [40]. Intriguingly, an independent mechanism achieves similar outcomes during EMT. TGFβ/Smad and MAP-kinase signaling transcriptionally induce expression of the salt-inducible kinase 1 (SIK1), a member of the AMP-regulated protein kinases; SIK1 associates with Smad7 and recruits Smurf2 to the TGFβ type I receptor, promoting its ubiquitination and lysosomal degradation [45,46]. Simultaneously, SIK1 associates with Par3 and promotes its phosphorylation and proteasomal degradation, thus facilitating the TGFβ-induced EMT [47]. These examples of Par3 and Par6 negative regulation by TGFβ signaling during EMT (Figure 2), suggest that in epithelial cells, tight junction disassembly is coupled to TGFβ receptor internalization and downregulation.

In addition to cell-cell contact remodeling during EMT, TGFβ promotes integrin receptor-ECM remodeling that facilitates cancer cell motility. During EMT, certain integrins are newly synthesized and other integrins are downregulated. Breast cancer cell EMT induced by TGFβ and metastasis require the presence of integrin-β1 [48]. Complementing integrin function, the adaptor hydrogen peroxide-inducible clone 5 (Hic-5) promotes RhoC small GTPase activity and focal adhesion assembly on mesenchymal cells, required for cell motility via invadopodia [49]. The function of Hic-5 is regulated by TGFβ, which upregulates its expression (possibly via Twist) but also promotes Hic-5 phosphorylation on tyrosine residues, catalyzed by the Src kinase [49]. The literature on regulation of integrin expression by TGFβ is extensive and only a few illustrative cases are summarized here.

### 2.3. Regulation of the Actin-Based Cytoskeleton during TGFβ-Induced EMT

As mesenchymal cells are generated by the EMT, ECM and cell adhesion remodeling processes are supported intracellularly by corresponding adaptations of the cytoskeleton, including all of its three major structural groups, microtubules, intermediate and microfilaments. We have already discussed the regulation of the RhoA small GTPase that controls actomyosin dynamics in association with tight junction remodeling (Figure 2). Further mechanisms feeding to the regulation of small GTPases during EMT include the transcriptional and miRNA-based regulation of the guanine exchange factor Net1 downstream of TGFβ (Figure 2) [50]. This is a good example of how complex signal transduction can be. TGFβ on one hand transcriptionally induces a cytoplasmic Net1 isoform with relatively rapid kinetics; sustained and relatively slower TGFβ signaling induces a nuclear Net1 isoform together with *miR-24*, which downregulates cytoplasmic Net1 [50]. This mechanism is thought to regulate distinct RhoA GTPase pools, a cytoplasmic pool possibly regulating tight junction loss during EMT (see above, Figure 2) and a nuclear pool of RhoA possibly mediating the cell cycle arrest of epithelial cells undergoing EMT, as established by independent studies [51,52]. TGFβ can additionally induce proteasomal degradation of two alternative guanine exchange factors of Rho GTPases during EMT, the leukemia-associated Rho guanine nucleotide exchange factor (LARG), and GEF-H1, a necessary mechanism for the adaptation of mesenchymal cells to integrin signaling mediated by ECM changes associated with cancer cell invasiveness [53]. Focal adhesions of mesenchymal cells generated through EMT are hubs of molecular reactions, such as the TGFβ-induced mobilization of the adaptor protein lipoma preferred partner (LPP) to focal adhesions, which crosslinks actin microfilaments to α-actinin and integrin complexes in order to facilitate breast cancer cell motility [54]. Similar mechanisms of actin microfilament-focal adhesion communication involve the adaptor protein moesin [55] and zyxin, the latter being transcriptionally induced by the Twist1 downstream of TGFβ signaling during the EMT [56]. Increased levels of zyxin regulate the ratio of functional focal adhesions and the resulting motility of lung cancer cells after the EMT [57]. The communication between ECM and actin microfilaments via focal adhesions is functionally dependent on the contractility mediated by myosins; TGFβ induces expression of myosin IIB by promoting alternative splicing of this protein isoform in breast cancer cells during EMT [58]. The expression of specific myosin isoforms during EMT is thought to contribute to differential patterns of cancer cell motility; accordingly, an ameboid movement can be induced in melanoma cells in response to TGFβ signaling via transcriptional cooperation of Smad2 and Cbp/p300-interacting transactivator 1 (CITED1) causing the expression of specific myosin light chain isoforms [59]. In most of these examples, motility mechanisms are considered to be activated once the cell–cell contacts are remodeled and the ECM-focal adhesion signaling networks have been stimulated.

### 2.4. TGFβ Controls the Expression of Many Other Growth Factors and Cytokines 

The EMT is a slow cellular process mediated via sequential waves of molecular activity. A large part of such an activity involves multiple growth factors and cytokines, which exhibit tissue-type specificity in various tumors [60]. We discuss some few examples here in order to illustrate this principle and consider cases where TGFβ is considered the starting point of the cytokine cascade (Figure 2). Crosstalk between TGFβ and the epidermal growth factor (EGF) is a well-documented case during breast cancer EMT. Accordingly, breast cancer cells undergoing EMT in response to TGFβ secrete EGF, which signals the activation of the focal adhesion kinase that mediates cytoskeletal remodeling that promotes cell motility [61]. The mitogen-inducible gene 6 (MIG6) is a negative regulator of the EGF receptor, which is under the negative control of *miR-200* in epithelial cells; when TGFβ induces EMT in lung and pancreatic cancer cells, it represses the expression of *miR-200*, indirectly resulting in the stabilization of MIG6 and a relative inhibition of EGF receptor [62]. Under such relative EGF receptor inhibition, TGFβ signaling is directed towards the Akt kinase, and gradually the cancer cells acquire resistance to EGF receptor inhibitors such as erlotinib, drug resistance being a phenomenon frequently linked to the process of EMT. Furthermore, TGFβ can induce the expression of Wnt family and sonic hedgehog (Shh) ligands in hepatocellular or lung adenocarcinoma cells; the action of Wnt or Shh is critical for the establishment of the EMT in these cancer cells under the influence of TGFβ [63,64]. A final case worth citing is the crosstalk between TGFβ and oncogenic Ras that is dependent on the downstream expression and signaling activity of the PDGF family together with the interleukin-like EMT-inducer (ILEI) (Figure 2). Transgenic mice expressing oncogenic K-Ras in their liver develop hepatocellular carcinoma and metastasis that is preceded by EMT enforced by coordinate signaling between K-Ras/MAP-kinase and TGFβ [65]. In such liver cancer cells, TGFβ promotes the translation of the *ILEI* mRNA and ILEI secretion [66], a pro-metastatic cytokine. In response to ILEI, liver cancer cells upregulate their PDGF receptors and downstream signaling via Stat3 and β-catenin, whose co-transcriptional complexes enforce stable mesenchymal cells with enhanced metastatic potential [66]. Using this mouse model, combinations of the PDGF receptor and TGFβ receptor inhibitors were proven effective in limiting the metastatic process, but not the single inhibitors [67], which highlights the modern trend in anti-cancer therapy based on the combinatorial treatment that targets multiple cooperating signaling pathways.

## 3. Regulation of EMT-TF Expression and Activity by TGFβ

As summarized above (Figure 2), the EMT-TFs can transcriptionally repress epithelial genes (e.g., *CDH1*) and induce mesenchymal genes (e.g., *Vimentin*, *fibronectin* (*FN1*), *N-cadherin* (*CDH2*)). In many models of cancer EMT, the high expression of one EMT-TF is accompanied by the induction of two or three additional EMT-TFs in a positive feed-forward manner. Once the contribution of EMT-TFs to EMT was elucidated, several reports established the ability of the same EMT-TFs to induce the generation of cancer stem cells (CSCs) [68]. Snail1 can be induced by TGFβ via Smad/MAP-kinase signaling that requires the early induction of the embryonic chromatin regulator high mobility group A2 (HMGA2), which then turns on Snail1 expression downstream of TGFβ [69,70]. Snail1 directly represses epithelial genes such as *CDH1* by forming complexes with Smads activated by TGFβ [71] and through the recruitment of lysine-specific histone demethylase 1 (LSD1/KDM1A) following LSD1-mediated H3K4 demethylation [72,73,74]. LSD1 physically associates with Snail1 through its Snail/Gfi-1 (SNAG) domain [73] and transcriptional repression can be regulated by the MOF (KAT8) acetyltransferase [75]. MOF acetylates LSD1 to reduce the association of LSD1 with epithelial gene promoters and thus inhibits the pro-EMT actions of Snail1 [75]. Ubiquitination is a dynamic post-translational modification, which is essential for the regulation of protein stability, signal transduction, and DNA repair. Snail1 activity is regulated by the ubiquitin-proteasome system through its phosphorylation by a glycogen synthase kinase 3β (GSK3β)-E3 ligase β-TrCP (β-transducin repeats-containing protein) cascade [76]. Conversely, the ubiquitin-editing enzyme A20, which is a key inflammatory and autoimmunity factor whose expression correlates with tumor aggressiveness, stabilizes Snail by mono-ubiquitination of specific Snail1 lysine residues, a mechanism that inhibits GSK3β-mediated Snail1 phosphorylation; as a result, A20 facilitates TGFβ-induced EMT in breast cancers [77]. 

Snail2/Slug can also repress several epithelial genes similar to Snail1. Transcriptional repression by Snail2/Slug is also regulated by epigenetic modifications. The Jumonji domain-containing protein 3 (JMJD3), a histone H3K27 demethylase, which is highly expressed in aggressive hepatocellular carcinoma cells, interacts with Smad3 [78], and catalyzes the transition of H3K27me^3^ and H3K27me^2^ to H3K27me^1^ on the *Snail2/Slug* promoter, switching the chromatin from a repressive to an active conformation. Consequently, Snail2/Slug is overexpressed and induces EMT [79]. In addition, Snail2/Slug is regulated by post-translational mechanisms during cell cycle progression. Snail2/Slug binds to the promoter of DNA synthesis and checkpoint-related genes, such as *Topoisomerase 1* (*TOP1*), *DNA ligase IV*, and *Rad17* to reduce cell proliferation and delay S-phase progression [80]. During the G1/S transition, Snail2/Slug is phosphorylated at Ser-54 and Ser-104 by cyclin E/cyclin-dependent kinase 2 (CDK2), whose activity is highest at the G1 to S phase transition, inducing the ubiquitination-proteasomal degradation of Snail2/Slug [80].

The chromatin silencing factor Bmi1, which is a member of the polycomb-repressive complex 1 (PRC1), is essential for Twist1-induced EMT [81]. Twist1 induces *Bmi1* through direct binding to its promoter, whereas Twist1 and Bmi1, in complex, bind to the *E-cadherin* promoter to induce EMT [81]. Twist1 activity is regulated by the protein kinase Akt1 [82], a member of the highly conserved Akt/PKB serine-threonine kinase family, known to promote tumor initiation and progression. Akt1 but not Akt2 or Akt3 interacts with and phosphorylates Twist1 at Ser-42, Thr-121, and Thr-123 to induce β-TrCP-mediated Twist1 degradation, and inhibit the EMT [82]. Conversely, some of the EMT-TFs are regulated by deubiquitination; ubiquitin chains can be removed from proteins by deubiquitinases (DUBs) also known as ubiquitin-specific protease (USPs). DUB3/USP17 deubiquitinates not only Snail2/Slug and Twist1 but also Smad4 [83] and so, accelerates the EMT by preventing ubiquitin-proteasomal degradation of these important EMT-TFs [84]. In addition, Twist1 can be stabilized by a different mechanism of ubiquitination. Really interesting new gene (RING) finger 8 (RNF8) is a RING finger E3 ligase and involved in DNA repair and telomere end protection, which ubiquitinates Twist1 through K63-linked poly-ubiquitination to sustain and stabilize Twist1 in the nucleus so that Twist1 can elicit the EMT [85]. Twist1 expression is transcriptionally regulated by the Nk2 homeobox 2.8 (Nkx2.8) transcription factor, which is a tumor suppressor; Nkx2.8 directly binds to the *Twist1* promoter region and transcriptionally represses its expression, subsequently inhibiting the EMT [86]. Conversely, SRY-related high-mobility-group box 5 (Sox5) facilitates Twist1-induced EMT transcriptionally; TGFβ induces Sox5 expression, which binds to the *Twist1* promoter and transcriptionally activates its expression to induce the EMT [87].

**Figure 3:**
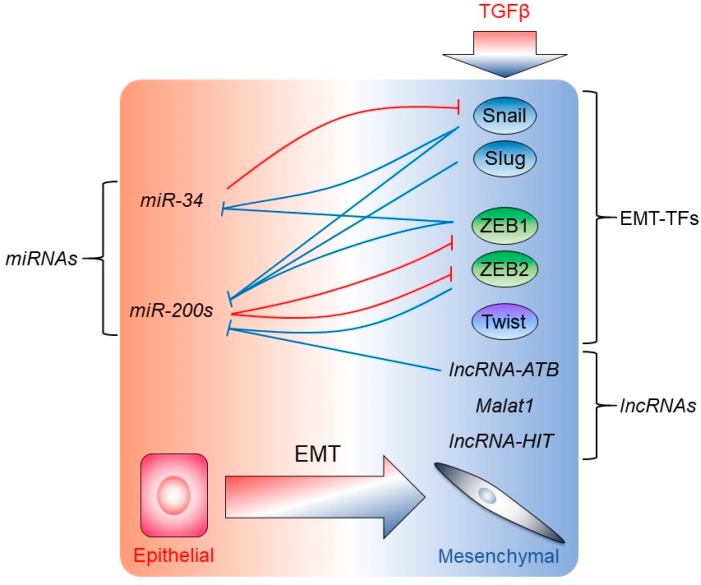
The non-coding RNAs regulate the EMT. Epithelial (left) and mesenchymal (right) genes are listed, the latter being transcriptionally induced by TGFβ signaling (thick arrow). Thin T-bars indicate the negative regulation of EMT-TFs by miRNAs and inversely, the negative regulation of miRNA expression by the EMT-TFs or *lncRNAs*. Negative transcriptional regulation is shown with blue T-bars, whereas negative regulation of mRNA translation and stability by miRNAs is shown with red T-bars.

It is well characterized that ZEB1 and ZEB2 directly repress several epithelial genes and form a double negative feedback loop with the *miR-200* family, which are classified as epithelial miRNAs (Figure 3). We discuss the relationship and regulation of EMT by miRNAs in the next section. ZEB1 and ZEB2 are transcriptionally regulated by Fos-related antigen 1 (Fra-1), which is a member of the Fos family of basic leucine zipper domain proteins, dimerized with c-Jun to form the activator protein 1 (AP-1), and negatively correlated with the prognosis of breast cancer patients [88]. Fra-1 binds to putative AP-1 sites in *TGFβ1* promoter regions to facilitate TGFβ signaling. Fra-1 also binds to putative AP-1 sites in the ZEB2 promoter and to an evolutionarily conserved region in intron 1 of ZEB1, increases ZEB1 and ZEB2 expression transcriptionally, and consequently activates TGFβ-induced EMT. Recent studies showed that the Polypyrimidine Tract Binding Protein 3 (PTBP3), which contains four RNA recognition motifs, is involved in miRNA-mediated gene decay and RNA splicing; PTBP3 binds to Argonaute 2 (AGO2), which is the main component of the RNA-induced silencing complex (RISC) and to the 3´ UTR of *ZEB1* to stabilize the *ZEB1* mRNA for EMT induction [89]. In addition to *miR-200s*, the epithelial splicing regulatory proteins (ESRPs) exert post-transcriptional regulation of ZEB1/2 (Figure 2). The ESRPs are identified as coordinators of the epithelial cell-specific splicing program, and ESRP1/2 are regulated by ZEB1/2 during TGFβ-induced EMT [90]. ZEB1 is regulated post-transcriptionally by the seven in absentia homolog (Siah) family, consisting of Siah1 and Siah2, which are evolutionarily conserved E3 RING finger ubiquitin ligases [91]. In breast epithelial tumors, Siah proteins are highly expressed and induce ubiquitination-proteasomal degradation of ZEB1; once the cells undergo EMT, Siah1/2 expression is decreased, but the mechanism of the decrease of Siah1/2 in mesenchymal cells has not yet been elucidated [91]. ZEB1 transcriptionally regulates ESRP1 expression, which is related to the switching of hyaluronan receptors, from the CD44v to the CD44s isoform [92]. ZEB1 directly binds to the *HAS2* promoter and induces HAS2 and hyaluronan synthesis; sequentially, hyaluronan activates the ZEB1 and CD44s expression, which further induces CD44s-mediated ZEB1 expression, proving the existence of a HAS2-hyaluronan-CD44s-ZEB1 positive feed-forward loop [92].

Additional post-transcriptional modifications can regulate TGFβ-Smad signaling and EMT-TF activities. One of these, SUMOylation, is a reversible process similar to ubiquitination, whereby three enzymes, the E1 activating enzymes SAE1/2, the E2 ubiquitin-conjugating enzyme 9 (Ubc9), and the E3 ligases such as the protein inhibitor of activated Stat (PIAS1-4), catalyze the binding of the small ubiquitin-related modifier (SUMO) to lysine residues on target proteins. SUMO and Ubc9 can interact with and induce SUMOylation of Smad4, consequently repressing the transcriptional activity of Smad4 and modulating TGFβ signaling [93]. ZEB2 interacts with the Cdc42 GTPase-activating protein (CdGAP), which is a major GTPase localized in the nucleus, and which can induce EMT by the direct repression of E-cadherin [94]. ZEB2 activity is also regulated by SUMOylation [93]. ZEB2 is SUMOylated at K391 and K866 by the Polycomb protein 2 (Pc2) SUMO E3 ligase, subsequently disrupting the recruitment of the corepressor C-terminal-binding protein (CtBP) to the *E-cadherin* promoter [95]. By combining the time-course EMT transcriptomic assays with the computational analysis of public cistronic data, three synergistic transcription factors ETS protooncogene 2 (ETS2), hepatocyte nuclear factor 4 (HNF4), and Jun protooncogene B (JUNB) have been identified as regulators of partial EMT, where cancer cells express both epithelial and mesenchymal markers (Figure 1) [96]. These regulators of partial EMT auto-regulate and positively regulate each other by feed-forward loops causing the repression of epithelial E-cadherin and induction of mesenchymal N-cadherin expression [96].

Compared to the EMT, the reverse process, MET is controlled by relatively unknown molecular mechanisms. Early reports showed that BMP signaling by regulating Id2 (Inhibitor of differentiation 2) expression can induce MET [97]. A hypothesis was put forward suggesting that Id proteins may counteract the function of basic helix-loop-helix EMT-TFs, i.e., Twist or E47, to enforce the MET, requires more direct testing. The Krüppel-like factor 4 (Klf4), which contributes to the generation of induced pluripotent stem cells, inhibits EMT and induces MET through direct interaction with the *E-cadherin* promoter [98]. Similar to Klf4, Klf10 transcriptionally represses Snail2/Slug expression through direct binding to its promoter and recruitment of histone deacetylase 1 (HDAC1) promoting the MET [99].

## 4. Regulation of EMT by miRNAs, lncRNAs, and mRNA Translational Mechanisms

Several reports have demonstrated that miRNAs play critical roles in cancer malignancy such as EMT and metastasis. The miRNAs are non-coding single-stranded small RNAs (21–25 nucleotides), post-transcriptionally repressing gene expression by sequence-specific interactions with the 3′-untranslated regions (UTRs) of cognate mRNA targets leading to mRNA degradation or inhibition of translation. Among those miRNAs related to the regulation of EMT/MET, the *miR-200* and the *miR-34* families are well characterized and exhibit tumor suppressor functions (Figure 3). The *miR-200* family consists of 5 miRNAs (*miR-200a*, *miR-200b*, *miR-200c*, *miR-141*, and *miR-429*), encoded by two polycistronic regions, the *miR-200b/miR-200a/miR-429* gene and *miR-200c/miR-141* gene on chromosome 1 and 12, respectively [100,101,102]. The *miR-200* family is highly expressed in epithelial cells and lower expressed in cells undergone the EMT [100,101,102]. The *miR-200* family forms a double-negative feedback loop with ZEB1/2 controlling the EMT plasticity, whereby *miR-200* downregulates ZEB1/2 in epithelial cells and ZEB1/2 repress the expression of *miR-200* in mesenchymal cells (Figure 3) [103]. In addition to ZEB1/2, the *miR-200* family expression is regulated by Snail1 (Figure 3). Snail1 exhibits bifunctional regulatory mechanisms: Snail1 directly represses *miR-200* family expression and induces EMT, conversely Snail1 binds to the *ZEB1* promoter region to activate its expression, and consequently, via ZEB1, Snail1 further represses the *miR-200* family expression [104]. Snail1, but also Snail2/Slug, repress the *miR-200* family expression through CpG methylation on the *miR-200* promoter region in dog kidney MDCK cells [105]. In addition to EMT-TFs, the *miR-200* family is also epigenetically regulated by fumarate, which is a component of the tricarboxylic acid cycle, metabolized by fumarate hydratase to malate [106]. It has been shown that fumarate inhibits α-ketoglutarate-dependent dioxygenases, which are important for histone demethylation [106]. Fumarate inhibits the expression of the *miR-200* family by promoting methylation of its promoter region to induce EMT [106]. The second epithelial *miR-34* family is also regulated by EMT-TFs (Figure 3). *miR-34* forms a double-negative feedback loop with Snail [107]. The *miR-34* binds to the 3′ UTR of the *Snail1* mRNA to repress its expression; on the other hand, not only Snail1 but ZEB1 can also bind to E-boxes in the *miR-34* promoter to repress its expression [107]. Further regulatory mechanisms among EMT-TFs and additional miRNAs are described below.

Apoptosis-stimulating protein of p53-2 (ASPP2) is a haploinsufficient tumor suppressor, which directly interacts with p53 family members and activates pro-apoptotic genes [108]. Hypoxia induces *miR-205*; *miR-205* directly binds to the 3′ UTR of the *ASPP2* mRNA to suppress its translation, subsequently inducing the EMT [108]. *miR-5003-3p* induces EMT through targeting two distinct mRNAs, one being mouse double minute 2 homolog (*MDM2*), the other being *E-cadherin* [109]. *miR-5003-3p* also stabilizes Snail1 protein expression through the direct targeting of MDM2, thus preventing the proteasomal degradation of Snail1 by MDM2, a mechanism that is independent of Snail1 phosphorylation by GSK3β [109]. In addition, *miR-5003-3p* directly represses the *E-cadherin* expression, simultaneously inducing the EMT [109]. The *miR-143* and *miR-145* are known as regulators of MAP-kinases, such as the mitogen-activated protein kinase kinase kinase 2 (MEKK2) [110]. MEKK2 stabilizes the transcriptional regulator TGIF via MAP-kinase-dependent phosphorylation, leading to the activation of TGFβ/Smad signaling [110]. In addition, these miRNAs play a role in the differentiation of vascular smooth muscle cells from neural crest stem cells and regulate cell motility through TGFβ/Smad-induced EMT and downregulation of tight junction proteins such as Zonula Occludens (ZO)-1/3 and Occludin [110]. The predicted targets of *miR-143* and *miR-145* regulate similar functional processes. One of them, CREB1, is the main transcriptional activator of Occludin and is predicted to also have a role in the transcription of ZO-1 [110]. *miR-22* has dual functions in cancer as a tumor suppressor and oncogene [111]. Several reports suggest that *miR-22* inhibits CREB1 and MYC pathways, through the targeting of CREB regulated transcription coactivator 1 (CRTC1), fms-related tyrosine kinase 3 (FLT3), and Myc binding protein (MYCBP), which are components of these pathways [111]. In addition, *miR-22* directly downregulates Snail1 and MAP-kinase 1 (MAPK1/ERK2). ERK2 is a positive regulator of Snail2/Slug activity, which then turns on the expression of Vimentin; thus, *miR-22* prevents the EMT by downregulating ERK2 and its downstream Sail2/Slug activity [111]. In prostate cancer, *miR-3622a* expression is inversely correlated to poor survival; *miR-3622a* expression is repressed by hypermethylation of its promoter region, which classifies *miR-3622a* as a tumor suppressor [112]. *miR-3622a* directly binds to the 3′ UTR of the *ZEB1* and *Snail2/Slug* mRNAs to inhibit the EMT [112]. *miR-373* is an oncogene that induces EMT; *miR-373* binds to the 3′ UTR of the *thioredoxin-interacting protein* (*TXNIP*) mRNA, an endogenous inhibitor of thioredoxin (Trx) [113]. Trx is important for generating reactive oxygen species (ROS), and its activity is negatively regulated by TXNIP binding to the redox-specific active cysteine residues of Trx [113]. The repression of TXNIP increases Trx activity and ROS levels and stabilizes the hypoxia-inducible factor (HIF) 1α. HIF1α directly binds to the *Twist1* promoter to induce Twist1 expression [113]. Twist1 also directly binds to the *miR-373* promoter, and as a result, *miR-373* activates the HIF1α-Twist1 pro-EMT axis through the repression of TXNIP [113]. *miR-27a* prevents the ubiquitin-proteasomal degradation of several EMT-TFs such as Snail1, Snail2/Slug, Twist1, and ZEB2 by the transcriptional repression of Fbxo45 [114]. Fbxo45 is a component of the Skp1-Pam-Fbxo45 complex, which is an atypical ubiquitin E3 ligase, binding to Snail1, Snail2/Slug, Twist1, and ZEB2 through its F-box and SPRY (SPla and the RYanodine Receptor) domains, respectively [114]. Several recent reports show that a hybrid EMT state is important for cancer metastasis as we described earlier (Figure 1); some miRNAs can regulate the hybrid EMT. For example, Twist1-induced *miR-424* through binding to the 3′ UTR of the *TGFB3* mRNA, induces an intermediate mesenchymal phenotype without obvious E-cadherin repression [115].

In addition to miRNAs, other non-coding RNAs play important roles as regulators of the EMT, including the long non-coding RNAs (lncRNAs), many of which are expressed under the regulatory input of TGFβ signaling (Figure 3) [116]. This is a rapidly growing area of research and here we summarize a few cases that illustrate important points of regulation. An established function of lncRNAs is to act as “sponges” that base-pair with various miRNAs, thus controlling the availability and half-life of these miRNAs. Thus, TGFβ induces the expression of *lncRNA-induced by TGFβ* (*lncRNA-ATB*) in liver cancer cells, which associates with and inactivates miRNAs of the *miR-200* family (Figure 3), promoting ZEB1/2 protein expression, once their mRNAs are released from the negative control exerted by the *miR-200* members [117]. *LncRNA-ATB* also regulates liver cancer metastasis beyond its impact on EMT, by positively affecting the expression of the IL-11 protein, an established cytokine with pro-metastatic actions, especially active in the TGFβ-enriched calcified bone tissue where breast or prostate cancer cells often metastasize [117]. In bladder cancer cells, when TGFβ induces the expression of the lncRNA known as *metastasis associated in lung adenocarcinoma transcript-1* (*Malat1*), the EMT is promoted because this lncRNA assembles into ribonucleoprotein particles together with the PRC2 component and histone methyltransferase suppressor of zeste 12 (Suz12), which thus mediates the well-known repression of E-cadherin [118]. Similar to the action of *Malat1* is the function of the *lncRNA-HIT* (*HOXa* transcript induced by *TGFβ*), which is transcriptionally induced by TGFβ signaling and participates in the mechanism of EMT by downregulating E-cadherin [116].

Most previous cases of miRNA action or lncRNA “sponges” are mechanistically based on the regulation of mRNA translation by ribosomes and the control of mRNA stability or decay. TGFβ signaling can affect mRNA translation through additional mechanisms that implicate protein-based regulation (Figure 2). For example, specific mRNAs contain in their 3′ UTRs conserved motifs that are recognized by trans-acting RNA-binding proteins that control the efficiency of the translation of these mRNAs. Such mRNAs include the cytokine ILEI discussed earlier, the TGFβ family member inhibin βA and the regulator of endocytosis and adaptor protein disabled-2 (Dab2) [119,120,121]. TGFβ signaling activates the Akt2 kinase, which phosphorylates the heterogeneous ribonucleoprotein E1 (hnRNPE1); this phosphorylation helps hnRNPE1 to dissociate from the translation elongation factor 1A1 (eEF1A1), which is then released and can promote the synthesis of ILEI or Dab2 protein [119,120,121]. Thus, binding of the hnRNPE1/eEF1A1 complex on *Dab2*, *inhibin βA* or *ILEI* mRNAs inhibits ribosomal subunit assembly necessary for driving protein synthesis. The three proteins discussed here positively mediate the EMT. It is anticipated that this mechanism may encompass the regulation of the synthesis of several proteins, since genome-wide screens performed by mRNA immunoprecipitation followed by sequencing of the mRNA [122] or by microarray analysis of mRNAs selected from the polysome-bound cellular pool [65] have identified a large number of translationally regulated mRNAs during TGFβ-induced EMT.

## 5. The Importance of EMT in Tumor Metastasis

Patient mortality due to primary tumor development is gradually decreasing thanks to technologies for early diagnosis and drugs with specific molecular targets. However, once a primary tumor metastasizes to distant organs, therapy based on surgery and anti-cancer drug treatment or radiation therapy become very ineffective. Therefore, 90% of cancer patients die of metastasis without effective treatment. For these reasons, the effective treatment and the elucidation of the mechanisms of metastasis are important [1,123]. Metastasis is a complex process involving the action of many cell types in addition to the malignant cells. Once a primary tumor acquires invasive power in a primary site, malignant cells invade the blood vessels (intravasation) [123]. After intravasation, tumor cells move to distant organs through the circulation or the lymphatic system; metastatic cells found in the blood circulation are known as circulating tumor cells (CTCs), and most of such CTCs are eliminated by the immune system, employing primarily dendritic cells and macrophages [123]. However, few of the CTCs can escape destruction by the immune system and metastasize to distant organs. Then, additional cells, including mature platelets, protect the CTCs from further destruction by the immune system and facilitate extravasation [124]. Finally, metastatic cells form secondary tumors at distant organs such as the brain, liver, and bone, in a manner that depends on the tissue origin of the primary tumor. 

Expression of the leucine-rich repeat-containing G protein-coupled receptor 4 (Lgr4) in human prostate cancer cell lines correlates with invasiveness and metastatic potential of these cells [125]. Studies in *Lgr4* knockout mice showed that prostate cancer development was delayed at an early age, but no effect on tumor formation and cancer cell proliferation could be observed later in their life [125]. In addition, lung metastasis and survival were attenuated in the *Lgr4* knockout mice. Ablation of Lgr4 in the prostate cancer cell line DU145 showed that migration, invasion, and EMT-TF expression were decreased, conversely, E-cadherin expression was increased [125]. Thus, Lgr4 plays an essential role in prostate cancer EMT and metastasis [125]. Triple-negative breast cancer (TNBC) is one of the most aggressive breast malignancies. Musculoaponeurotic fibrosarcoma (MAF) oncogene family protein K (MAFK), a member of the small MAF family of transcription factors, is highly expressed in TNBC, due to induction by TGFβ [126]. MAFK promotes EMT through transcriptional induction of the transmembrane glycoprotein nmb (GPNMB) and directly represses E-cadherin expression, subsequently affecting tumor formation, migration, invasion, and metastasis [126]. Lysyl oxidase-like 2 (LOXL2), which is a member of the lysyl oxidase family, and E47, which is one of the basic helix-loop-helix EMT-TFs, interact with each other and directly repress the *E-cadherin* gene to induce EMT [127]. In addition, LOXL2 and E47 directly regulate *FN1* and cytokines such as the tumor necrosis factor-α (TNFα) and the granulocyte-monocyte colony stimulating factor (GM-CSF), which are required for the formation of the pre-metastatic niche [127]. A screen performed to identify competing endogenous RNAs (ceRNAs) that govern metastasis revealed that the targets of the tumor suppressor *miR-181b* are integrin α1 (ITGA1) and adenylyl cyclase 9 (ADCY9) [128,129]. At basal levels of expression, *miR-181b* represses both *ITGA1* and *ADCY9* mRNAs and inhibits the metastatic cascade. However, once ZEB1 induces *ITGA1* expression, the high levels of the *ITGA1* mRNA preferentially act as a ceRNA (“sponge”) for the *miR-181b*; then ADCY9 protein synthesis is de-repressed and ADCY9-produced cAMP promotes the metastasis cascade [128,129]. Knockout of two EMT-TFs, Snail1 and ZEB1 from metastatic TNBC cells using the CRISPR/Cas9 system revealed a partial reversion towards an epithelial phenotype (MET) and the impact on these two factors on cell proliferation and motility [130,131]. These studies strongly suggest that aggressive cancer cells that express multiple EMT-TFs have undergone a long history of EMT-related change. Reversion to a progenitor epithelial cell type may require the removal of all EMT-TFs expressed in a given tumor cell.

The fact that EMT is related to and important for migration, invasion, and metastasis (Figure 1) is supported by many studies [1]. However, reports of carefully designed genetic models in mice have demonstrated that EMT is not required for cancer metastasis but is important for chemoresistance development in pancreatic and breast cancer mouse models [132,133]. Using transgenic EMT reporter mice, which is a triple-transgenic breast cancer mouse model (*MMTV-PyMT/Rosa26-loxP-RFP-loxP-GFP/Fsp1-Cre*) with the following features: the oncogene Polyoma Virus middle T antigen under the direction of the mouse mammary tumor virus (MMTV-PyMT) causes breast tumorigenesis; the Cre-switchable fluorescent marker (*loxP-RFP-loxP-GFP*) is ubiquitously expressed by the *β-actin* promoter of the *Rosa 26* locus, whereas the *fibroblast-specific protein 1* (*Fsp1*) promoter drives Cre recombinase expression, causing the loss of RFP and switch on of GFP only in cells that express Fsp1, i.e., mesenchymal cells [132]. Accordingly, epithelial cells express RFP under the control of the *β-actin* promoter. Once the cells undergo EMT, the *Fsp1* promoter is activated in the early EMT stage and induces Cre recombinase expression, subsequently genetically deleting the RFP cassette. As a result, the fluorescent protein expression switches from RFP to GFP permanently [132]. In other words, once the cells have undergone EMT, they express GFP sustainably. In the EMT reporter mice, spontaneous primary breast tumors were developed and gave rise to lung metastases [132]. The primary breast tumor cells expressed both RFP and GFP, but metastatic tumor cells did not express GFP [132]. This suggested that the metastatic cells did not have to undergo EMT in this model system [132]. In agreement, an independent study used conditional Snail1 and Twist1 knock-out in the pancreatic epithelium of mice, which could spontaneously develop primary pancreatic tumors and metastases to the lung, liver, and spleen due to their Pdx1-Cre-mediated activation of K-Ras and mutant p53 (KPC model) [133]. The knock-out mice showed similar results as the EMT reporter mice, as pancreatic cancer did not show signs of EMT in the metastatic tumors; however, the EMT suppressed the expression of drug transporters, including the equilibrative nucleoside transporter 1 (ENT1) and the concentrating nucleoside transporter 3 (Cnt3), which assisted the metastatic tumor cells to escape from the injection of anti-cancer drugs used to treat the mice [133].

On the other hand, in contrast to the Snail1 and Twist1 knock-outs, ZEB1 knock-out suppressed the metastatic dissemination of primary tumor cells using the same KPC pancreatic cancer model [134]. These reports necessitate a deeper understanding of the contribution of EMT to metastasis, suggesting that the relationship between EMT and metastasis is contextual [135,136]. Another case where relatively short distance metastasis that may not necessitate the EMT is when colorectal cancer cells infiltrate and establish metastatic nodules to the peritoneum [137]. Small masses of intestinal epithelial cells disseminate from the growing intestinal tumors in the form of shed epithelial spheres that retain the polarity and architectural organization of the normal tissue [137]. As the tumors disseminate these metastatic outgrowths that pinch off the hyperplastic epithelium, invasive spheroids appear as if the tumor cells have “reverted” their polarity, in other words, they invade by generating spheres made of cells with their apical membranes in the periphery of the sphere, possibly because no basal membrane components are engulfed into the tumor spheres [137]. Such tumor spheres can generate daughter tumor spheres during infiltration of the surrounding peritoneal cavity as a means of propagating their metastatic potential. Unexpectedly, this study proposed that “weak” TGFβ signaling is required for the generation of tumor spheroids via budding and for the infiltrative dissemination of these tumors [137]. The meaning of “weak” TGFβ signaling and the mechanism by which tumor tissue delamination is regulated by signaling molecules downstream of TGFβ remain to be understood. 

A possible explanation for some of the controversial findings regarding the impact of EMT on cancer metastasis can be the fact that experimental models usually analyze the impact of “end-stages”, i.e., the complete loss of an EMT-TF or high overexpression of an EMT-TF. The new emphasis of an old hypothesis [4], the “metastable” or “hybrid EMT” or “partial EMT” (Figure 1) recently gained some attention and proposed at least partial solutions to the previously analyzed problems [6,7]. Recently, specific cell surface markers identifying the hybrid EMT have been reported. After screening 176 cell surface markers, three markers of hybrid EMT—CD51 (Integrin α_v_), CD61 (Integrin β_3_) and CD106 (vascular cell adhesion molecule-1, VCAM-1)—were identified [138]. Hybrid EMT can be divided into different states with different patterns of expression of these cell surface markers. In strictly epithelial cells, cancer cells express the typical epithelial marker EpCam without the three above-cell-surface markers [138]. In the early state of EMT, the cells lose EpCam expression with/without CD106 expression (Figure 1). Interestingly, this early EMT state of the cells is thought to be most important for providing metastatic ability [138]. Forward to a more mesenchymal state, the cells will express CD51 and CD61 (Figure 1). In their most mesenchymal state, the cells become triple positive for CD51, CD61, and CD106 expression [138]. Xy et al. established a breast tumor-specific Twist1 knock-out mouse model and showed that Twist1 did not affect tumor initiation and primary growth but affected the tumor cell dissemination and metastasis [139]. Twist1-positive cells co-expressed other EMT-TFs, such as Snail1, Snail2/Slug, and ZEB2, and despite this, showed a partial EMT phenotype, which is E-cadherin and Vimentin double-positive. This report also supports the model whereby Twist1-induced partial EMT is important for metastasis [139]. The real picture probably involves a variety of different dissemination scenarios for tumor metastasis; some of these scenarios are clearly based on EMT whereas other scenarios rely more on a partial EMT or not at all on an EMT, suggesting that it will be important to classify tumor metastasis based on the ability of tumor cells to rely or not rely on EMT. Furthermore, partial or hybrid EMT is a concept that may represent the ability of modern technology to detect intermediate phenotypes based on the expression of epithelial and mesenchymal proteins in one and the same cell. Whether such hybrid cells are the key mediators of a specific biological process, such as metastasis in vivo remains unclear. Correlations made though so far provide some strong clues for the importance of a hybrid phenotype. It is also worth considering that hybrid E/M cells may be the source of more mesenchymal or more epithelial cells, thus generating heterogeneous populations throughout the long trajectory of metastasis. In the latter dynamic case, it may be impossible to establish cause and effect relationships with specific cell types and the process of metastasis. Rather, correlations of a specific stage in the metastatic cascade and a certain expression pattern of genes and proteins in cells accumulating during this specific stage may be the best that can be achieved by this type of exciting modern cancer research.

In addition to examples where TGFβ-induced EMT or even hybrid EMT may contribute to cancer metastasis, the metastasis literature provides several cases for diverse roles of TGFβ, which are possibly independent of the process of EMT [21,140]. Here we briefly summarize a few such cases of tumor metastasis that illustrate diverse mechanisms of action of TGFβ signaling based on the role of different cell types in the tumor microenvironment. It is well known that Id1 is induced by BMP signaling, but TGFβ can also induce Id1 in human breast cancer cells [141]. Id1-overexpressing human breast cancer cells (HMLE) showed high tumor-initiating capacity (TIC) and E-cadherin without Vimentin expression (MET). On the other hand, Twist-overexpressing HMLE cells showed TIC but decreased E-cadherin and increased Vimentin expression (EMT). These data suggest that Id1 can induce a TIC phenotype in HMLE cells independently of the induction of EMT [141]. In addition, pulmonary metastases showed a positive correlation between Id1 and epithelial phenotype [141]. Snail or Twist-overexpressing HMLE cells showed the EMT phenotype; combining with Id1 overexpression, Twist-overexpressing cells switched back to the epithelial phenotype (MET), but not Snail-overexpressing cells in vitro and in vivo [141]. These data suggest that TGFβ-induced Id1 expression is important to facilitate MET and promote metastatic colonization in Twist- but not Snail-expressing cells. This is a very good example of what is often referred to as the contextual role of EMT during metastasis.

Several reports suggest that TGFβ signaling is silenced by several mutations in colorectal cancers (CRCs), but a TGFβ type I receptor kinase inhibitor prevents CRC metastasis in a mouse model system [142]. By focusing on the contribution of the tumor microenvironment to metastasis, CAFs were identified to secrete TGFβ, which induced IL-11 expression, followed by the activation of GP130/STAT signaling, ending with an enhanced metastatic initiation [142].

Osteolytic bone metastasis from human breast and pancreatic cancer is also inhibited by TGFβ type I receptor kinase inhibitors [143,144]. In the target bone tissue, breast carcinoma cells that extravasate from the bone marrow fenestrated vessels, respond to stromal TGFβ and turn on the expression of IL-11 and Parathyroid Hormone-Related Protein, which act on osteoblasts and mobilize the secretion of the cytokine RANKL, which finally leads to osteoclast differentiation and activity [143]. The activated osteoclasts destroy mature bone tissue generating a new niche for the metastatic cells [143]. Indirectly, osteoclast activity releases bone matrix-deposited growth factors including TGFβs and BMPs, which then act on the breast cancer cells and promote a continuous and vicious cycle of signaling that establishes the productive metastatic colonization and secondary tumor growth [143]. By screening for TGFβ-regulated genes in prostate cancer cells, the most highly upregulated gene found was *PMEPA1* (prostate transmembrane protein androgen induced-1) [144]. PMEPA1 has three isoforms, PMEPA1a/b/c, PMEPA1a/b containing a membrane-bound domain, and PMEPA1c being cytosolic. Only the membrane-bound domain containing PMEPA1a/b negatively regulated TGFβ signaling through the recruitment of Smad2/3 and E3 ubiquitin ligases, but the inhibition was not dependent on ubiquitin-proteasomal degradation [144]. In bone metastatic sites, the *PMEPA1* gene promoter is epigenetically silenced by methyltransferases and, as a result, TGFβ signaling is enhanced and assists in the bone metastasis of the pancreatic cancer cells [144]. Another report also suggests that TGFβ primes human breast cancer cells to lung metastasis through the activation of Angiopoietin-like 4 (ANGPTL4) which disrupts endothelial cell-cell junctions, thus permitting metastatic breast cells to extravasate and initiate metastatic colonies in the lung [145].

Finally, tumor-derived TGFβ plays key roles in the regulation of the immune system within the tumor microenvironment. Multiple such examples exist [21,140]. An exceptional case that illustrates the diversity of actions TGFβ can take during metastasis is exemplified by studies of the knockout of the *TGFβ type II receptor* gene in breast carcinomas, which become refractory to TGFβ responses, yet, they oversecrete TGFβ and chemokines of the CXCL family (SDF-1, CXCL5), which act as chemo-attractants and recruit myeloid cells to the tumor [146]. These myeloid cells then take an active role in helping the invasion and metastasis of the breast carcinoma cells [146].

It is worth noting that in many of the above examples, the mouse models used that exhibit high dependency on TGFβ signaling also depend on highly aggressive tumor cells, often those with mesenchymal properties (e.g., MDA-MB-231 breast cancer cells or CAFs). Thus, it is not easy to separate TGFβ-regulated metastasis from the mesenchymal fate of tumor cells. Possibly the examples where TGFβ acts on immune cells may be independent of the effects on EMT, however, even in these cases, the reported experiments measure the expression of mesenchymal markers on the metastatic tumor cells.

Furthermore, modern studies on EMT, epithelial cell polarization, and invasive cell growth are much assisted by the advent of 3D cultures that grow in suspension, in a defined ECM, such as basement membrane-like laminin-enriched matrix or growth factor-enriched matrix (matrigel) or in specially fabricated microfluidic chambers [147,148,149,150]. In 3D, tumor cells build a more physiological tissue architecture and signals emanating from growth factors like TGFβ generate molecular circuits that are much closer to an in vivo situation [148,150]. Furthermore, the development of modern microscopic technologies that operate in a non-invasive manner, and the use of the second or third harmonic generation principle, allow for the clear visualization of ECM components, such as collagens, and the direct attachment and movement of tumor cells during invasive growth in vivo [148,151,152]. Such technology, combined with intravital microscopy that permits observation in tumor tissue in the absence of surgery, and in various depths from the skin, provides the unique opportunity to uncover new mechanisms of tumor cell behavior during metastasis [152,153]. It is worth mentioning that the pioneering work on intravital microscopy of metastatic dissemination did indeed observe single-cell invasion and intravasation into blood vessels, a phenotypic feature fully compatible with the process of EMT [152]. However, a combination of intravital microscopy and 3D organoid cultures with a cell-specific marker analysis in Her2^+^ breast cancer cells revealed strong evidence for a hybrid E/M phenotype of early disseminating metastatic cells [154]. An independent technology known as CUBIC is based on the stripping of fat tissue from the whole body of a mouse after formalin-based fixation, providing the unique opportunity of analysis of single cells in every organ of the animal [155]. This method of quantitative analysis with single-cell resolution also provided evidence for a positive role of TGFβ-mediated EMT in cancer metastasis [155]. All these technological advances offer unique opportunities for the classification of different tumor metastasis patterns of dissemination, clarifying the instances under which, full EMT, hybrid or partial EMT and single-cell or collective cell invasion are the mechanisms of metastatic spread. Future experiments may combine fate mapping of tumors in mouse models with non-invasive microscopy coupled to single-cell gene expression analysis to reveal unequivocally the reasons why many carcinomas choose the EMT pathways in order to progress into metastasis. In addition, these technologies provide a new means of testing novel drug combinations in a setting that is closer to in vivo case compared to the traditional 2D cell culture on a plastic dish.

## 6. New Approaches Towards the Treatment of Metastasis

Based on all previous discussion, it is logical to think that genetic, protein-based or chemical means that can inhibit the EMT may be a good approach towards the prevention of metastasis. Accordingly, if EMT signals are inhibited, one would expect MET to be induced in cancer cells and an anticipated lower rate of metastasis. However, pioneering studies inspecting the in vivo relevance of MET in mouse models have already suggested that CTCs in which the EMT is partially blocked, exhibit facilitated colonization of distant organs [156,157]. The details of the regulation of hybrid EMT remain largely unknown but gradually become elucidated. In this context, if the early state of hybrid EMT (EpCam negative, CD106 positive cancer cell population [138]), is important for metastatic ability in human malignancies, then approaches targeting this group of cells will have a strong impact in cancer therapy. Focusing on the hybrid E/M-specific cell surface markers (CD51, CD61, and CD106, [138]), and learning more details about the regulation of these cell surface proteins and their functions, might be useful for the development of new diagnostic tools and for the treatment of cancer metastasis. The early EMT phenotype has also been linked to a relative downregulation of two proteasomal subunits (β2 and β5) as if mesenchymal transition requires lower proteolytic activity in the cell [158]. In agreement, specific proteasome inhibitors with currently active trials as agents against human cancer enhance the EMT response and the pool of breast cancer stem cells, suggesting caution in the use of these inhibitors in cancer therapy [158].

The role of TGFβ as an inducer of EMT has also brought this pathway in central focus in the anti-cancer drug arena. For example, combinations of highly specific inhibitors targeting the TGFβ receptor kinase with cytotoxic drugs, such as paclitaxel, effectively blocked the EMT in breast cancer cells and their metastatic potential to colonize the lung [159]. The basic understanding of translational mechanisms that activate pro-EMT factors in response to TGFβ led to the chemical inhibitor 4Ei-1, which locks the activity of the translation factor eIF4E [160]. 4Ei-1 can inhibit TGFβ-induced EMT primarily because of a drastic reduction in the *Snail1* mRNA pool that remains ready for protein synthesis on polysomes, a characteristic phase of an early EMT response [160]. Based on extensive crosstalk between TGFβ signaling and the tumor suppressor p53, studies using nutlin-3, an established stabilizer of p53 and enhancer of its transcriptional activity, indicated that nutlin-3 can inhibit TGFβ-induced EMT in carcinoma cells; unexpectedly, nutlin-3 reduced the phosphorylation of Smad2 and Smad3 and suppressed Snail1/Snail2 induction by TGFβ [161]. This study raised the concern that nutlin-3 may have novel functions by possibly targeting the TGFβ receptor kinases, which remains to be investigated. On the other hand, the previously established negative role of wild-type and mutant p53 on TGFβ-induced EMT [162] is fully compatible with the studies of nutlin-3 [161]. Another inhibitor of breast cancer cell EMT and Snail1 upregulation induced by TGFβ is disulfiram, which blocks NF-κB signaling, and which was used to treat organ damage caused by alcohol consumption [163]. How disulfiram inhibits the EMT remains to be analyzed deeper, as well as its potential use as an anti-metastatic drug. In addition, drugs targeting chromatin modifications show promise as anti-EMT agents. The histone deacetylase inhibitor vorinostat could decrease phenotypic changes associated with TGFβ-mediated EMT in vitro and reduced the metastatic potential of biliary tract cancer cells [164]. This chemical inhibitor also reverted to some extent the resistance to cytotoxic therapy that such biliary malignant cells develop [164]. An exciting side observation has indicated that vorinostat reduced the pools of nuclear Smad4 downstream of TGFβ [164], which is compatible with an acetylation-dependent mechanism of Smad4 export from the nucleus [165].

A rather intense recent activity analyzes the anti-metastatic potential of natural compounds abundant in herbs or recipes of classical alternative medicine [166]. One such compound, curcumin, already used to treat malignancy, can shift thyroid cancer cells towards an epithelial phenotype presumably because of a yet uncharacterized anti-TGFβ mode of action possibly targeting TGFβ receptor expression and activity [167]. In liver cancer, EMT induced by TGFβ can be counteracted by sulforaphane, a natural compound abundant in various vegetables, which can reduce ROS levels, raising the possibility of its usefulness as an anti-metastatic compound in addition to its established anti-tumorigenic action [168]. Thus, attempts to generate specific inhibitors of EMT, possibly bypassing the danger of accumulating MET-dependent pro-metastatic side effects, will continue actively, as they carry good potential and promise for the development of clinically useful anti-metastatic drugs.

## 7. Concluding Remarks

The mechanisms that drive the EMT in tumor cells are investigated in greater depth and become gradually understood. Recent efforts to elucidate complete signaling pathways, the role of post-translational modifications of diverse proteins that control the EMT and even non-coding RNAs that synergize with proteins to provide more quantitative control of EMT generate exciting new principles of fundamental cell biological processes. Yet, more remains to be discovered with respect to the process of MET (Figure 1). Although MET is logically thought as a mere reversion of the EMT, the now established concept of partial EMT together with the molecular complexity that governs the step-wise transitions in differentiation propose an equally complex mechanism of MET progression such as the one that initiates the EMT, which is worth elucidating in full detail. More important from the point of view of cancer research remains the clarification and detailed elucidation of the circumstances under which the EMT contributes to the early stages of metastatic dissemination of malignant cells. It is equally necessary to explain to what extent molecular mechanisms of EMT that generate cancer stem cells that promote chemoresistance and favor metastasis, represent distinct processes that may have as common features the action of key molecules, such as specific EMT-TFs, yet are orchestrated by unique sequences of interactions and regulatory steps. An equally important question to resolve is the extent to which the factors that drive the EMT, and whose expression is misregulated in human tumors, as clearly established by transcriptomic screens of many carcinomas and even non-epithelial malignancies, remain expressed in metastatic tumor cells by necessity to sustain a long-term (possibly hybrid E/M) mesenchymal cell. Can the removal of these factors, e.g., Snail1 or ZEB1 or combinations, revert aggressive tumor cells to a less malignant, adenoma-like phenotypic state? Discoveries on mechanisms of differentiation changes during malignant progression are a fruitful territory for the development of more advanced diagnostics and drugs that can silence metastatic potential in cancer.

## Abbreviations

4Ei-1	eIF4E inhibitor 1
ADCY9	adenylyl cyclase 9
AGO2	argonaute 2
Akt (1, 2, 3)	oncogene Akt serine/threonine kinase 1, 2, 3 (alias: protein kinase B, PKB)
AP-1	activator protein 1
ASPP2	apoptosis-stimulating protein of p53-2
ANGPTL4	angiopoetin-like 4
BMP	bone morphogenetic protein
CAF	cancer-associated fibroblast
CD51	cell differentiation 51, alias: integrin α_v_
CD61	cell differentiation 61, alias: integrin β_3_
CD106	cell differentiation 106, alias: vascular cell adhesion molecule-1, VCAM-1
CdGAP	Cdc42 GTPase-activating protein
CDH1	E-cadherin
CDH2	N-cadherin
CDK2	cyclin-dependent kinase 2
ceRNA	competing endogenous RNA
CITED1	Cbp/p300-interacting transactivator 1
Cnt3	concentrating nucleoside transporter 3
CpG	cytosine-phosphate-guanine nukleotide
CRB3	Crumbs3
CRC	colorectal cancer
Cre	cyclization recombinase
CREB1	cAMP-responsive element binding protein 1
CRTC1	CREB regulated transcription coactivator 1
CSC	cancer stem cell
CtBP	c-terminal-binding protein
CTC	circulating tumor cell
CUBIC	clear, unobstructed brain/body imaging cocktails and computational analysis
CXCL	C-X-C motif chemokine ligand
Dab2	disabled-2
DUB	deubiquitinase
ECM	extracellular matrix
eEF1A1	eukaryotic elongation factor 1A1
EGF	epidermal growth factor
EMT	epithelial-mesenchymal transition
EMT-TFs	EMT transcription factors
EndMT	endothelial-mesenchymal transition
ENT1	equilibrative nucleoside transporter 1
EpCam	epithelial cell adhesion molecule
ERK2	extracellular signal regulated kinase 2
ESRP	epithelial splicing regulatory protein
ETS2	ETS proto-oncogene 2
Fbxo45	F-box homologue 45
FLT3	Fms-related tyrosine kinase 3
FN1	fibronectin 1
Fos	FBJ osteosarcoma oncogene
Fra-1	Fos-related antigen 1
Fsp1	fibroblast-specific protein 1
GEF-H1	guanine exchange factor H1
GFP	green fluorescent protein
GM-CSF	granulocyte-monocyte colony stimulating factor
GPNMB	glycoprotein nmb
GSK3β	glycogen synthase kinase 3β
H3K4	histone 3, lysine 4
H3K27me	histone 3, lysine 27, methylation
HAS2	hyaluronan synthase 2
HDAC1	histone deacetylase 1
Hic-5	hydrogen peroxide-inducible clone 5
HIF	hypoxia-inducible factor
HMGA2	high mobility group A2
HMLE	human mammary epithelial cells immortalized with the large T antigen
HNF4	hepatocyte nuclear factor 4
hnRNPE1	heterogeneous ribonucleoprotein E1
Id1/2	inhibitor of differentiation 1/2
IL-11	interleukin 11
ILEI	interleukin-like EMT-inducer
I-Smad	inhibitory Smad
ITGA1	integrin α_1_
JMJD3	Jumonji domain-containing protein 3
JNK	Jun N-terminal kinase
Jun	Jun proto-oncogene
Klf4	Krüppel-like factor 4
KPC	K-Ras and mutant p53 pancreatic carcinoma mouse model
LARG	leukemia-associated Rho guanine nucleotide exchange factor
Lgr4	leucine-rich repeat-containing G protein-coupled receptor 4
lncRNA	long non-coding RNA
lncRNA-ATB	lncRNA-induced by TGFβ
lncRNA-HIT	HOXa transcript induced by TGFβ
LOXL2	lysyl oxidase-like 2
LSD1	lysine-specific histone demethylase 1, alias: KDM1A
MAF	musculoaponeurotic fibrosarcoma
MAFK	MAF oncogene family protein K
Malat1	metastasis associated in lung adenocarcinoma transcript-1
MAP-kinase	mitogen-activated protein kinase
MAPK1	MAP-kinase 1
MDM2	mouse double minute 2 homolog
MEKK2	mitogen-activated protein kinase kinase kinase 2
MET	mesenchymal-epithelial transition
MIG6	mitogen-inducible gene 6
MiRNA	micro-RNA
MMP	matrix metalloprotease
MMTV-PyMT	mouse mammary tumor virus polyoma virus middle T antigen
MOF	males absent on the first (alias: KAT8, lysine acetyltransferase 8)
MT1-MMP	membrane type I-matrix metalloprotease
MYCBP	Myc binding protein
Net1	neuroepithelial cell transforming 1
NF-κB	nuclear factor-κB
Nkx2.8	Nk2 homeobox 2.8
PAI-1	plasminogen activator inhibitor 1
Par3/6	partitioning-defective 3/6
Pc2	polycomb protein 2
PDGF	platelet-derived growth factor
PIAS	protein inhibitor of activated Stat
PMEPAI	prostate transmembrane protein androgen induced-1
PRC1/2	polycomb-repressive complex 1 and 2
PTBP3	polypyrimidine tract binding protein 3
R-Smad	receptor-activated Smad
Rad17	radiation-induced 17 (alias: checkpoint clamp loader component)
RFP	red fluorescent protein
Rho	Ras homologue
RISC	RNA-induced silencing complex
RING	really interesting new gene
RNF8	RING finger 8
ROS	reactive oxygen species
RTK	receptor tyrosine kinase
SAE1/2	SUMO1 activating enzyme subunit 1
Shh	sonic hedegehog
Siah	seven in absentia homolog
SIK1	salt-inducible kinase 1
Smurf	Smad ubiquitylation regulatory factor
SND1	staphylococcal nuclease and tudor domain containing 1
SNAG	Snail1/Gfi-1
Sox5	SRY-related high-mobility-group box 5
SPRY	SP1a and the Ryanodine Receptor
Src	(Rous) sarcoma (virus oncogene)
STAT	signal transducer and activator of transcription
SUMO	small ubiquitin-related modifier
Suz12	suppressor of zeste 12
TAK1	TGFβ-activated kinase 1
TAZ	transcriptional coactivator with a PDZ-binding domain
TF	transcription factor
TGFβ	transforming growth factor β
TGIF	TGFβ-induced factor homeobox 1
TIC	tumor initiating cell
TNBC	triple-negative breast cancer
TNFα	tumor necrosis factor-α
TOP1	topoisomerase 1
TRAF	TNFα receptor-associated factor
β-TrCP	β-transducin repeats-containing protein
Trx	thioredoxin
TXNIP	thioredoxin-interacting protein
UBC9	ubiquitin-conjugating enzyme 9
USP	ubiquitin-specific protease
UTR	untranslated region
Wnt	wingless and integration site
YAP	Yes-associated protein
ZEB1/2	zinc finger E-box binding homeobox 1/2
ZO	zonula occludens
